# Towards consilience in science of learning: data as currency for collaboration

**DOI:** 10.1038/npjscilearn.2016.9

**Published:** 2016-06-08

**Authors:** Soo-Siang Lim

**Affiliations:** 1 National Science Foundation, Arlington, VA, USA

Learning and knowledge are key drivers of human progress. We face renewed urgency for effective education and lifelong learning to meet the demands of knowledge-based, technology-driven economies and labour markets of today.

The topic of learning has motivated research in many disciplines. However, knowledge about learning is often not shared or integrated across disciplinary boundaries, and is confined by language, modes of analysis and standards of validation within the theories, assumptions, concepts of each discipline. Such artifacts of scholarship stand in the way of a deeper and holistic understanding of learning with all its complexities.

To address these challenges, the US National Science Foundation (NSF) established the Science of Learning Centers Program in 2003^1^ to advance fundamental knowledge about learning through integrative and interdisciplinary research; and to connect the research to societal challenges, including education, technology innovation and workforce challenges. Six Science of Learning Centers were established in 2004 and 2006.

The Science of Learning, as envisioned by NSF, builds on a long history of research on learning in diverse disciplines including biology, neuroscience, cognitive and behavioural sciences, social science, mathematics, computer and information sciences and education. The Science of Learning promotes integration of knowledge across disciplines and many levels of analysis through shared conceptualizations that anchor new experimentation and explanation. A goal of such interdisciplinary endeavours would be consilience, or the ‘linking of facts and facts-based theory across disciplines’ to create unity of knowledge.^2^

Research on learning lends itself naturally to exploration of how the findings might inform education policy and practice, and conversely, how knowledge and experiences of practitioners can inform research agendas and theory building. The pursuit of consilience in the science of learning must by necessity include not only the researchers in the science and education communities, but also education practitioners and policy makers. As governments grapple with the best policies to educate their citizenry, how wisely the policy is chosen depends on the ease with which knowledge from available science and continuing discoveries will be transferred to and used by the practitioner and policy communities. Can scientists and other expert communities agree on a common body of principles and evidentiary proof about how people learn? Is consilience possible?

The explicit call for integrative, interdisciplinary approaches to learning, as exemplified by the NSF Science of Learning Centers Program could be a move towards consilience. This has been followed by a groundswell of similar initiatives elsewhere, including the establishment of the Australian Science of Learning Centre at University of Queensland (2013), Hong Kong University’s Science of Learning Initiative (2013), the Brazilian Network of Science for Education (2015, Federal University of Rio de Janeiro) and the Science of Learning initiative at the Singapore National Research Foundation (2015). In the US, independent of the 6 NSF-funded Science of Learning Centers, a Science of Learning Institute was established at Johns Hopkins University (2013), and a Science of Learning Center at the University of Chicago (2015).

Other efforts among many to bridge the existing disconnects between research and education practice include several publications^3–9^ about the most productive ways forward. These discussions have been continued through many venues, including the Latin American School for Education, Cognitive and Neural Science,^10^ the International School on Mind, Brain and Education in Erice, Italy,^11^ and the International Mind, Brain and Education Society (IMBES).

These many convergences of interest in learning from multiple sectors, combined with the exponential increase in data and information about learning, are offering unprecedented opportunities for progress towards consilience in science of learning. Consilience can only be arrived at through collaboration, and I posit here that successful collaboration to this end require that we change our thinking about data as things to be stored and occasionally retrieved, to data as currency for meaningful collaborations and meaning-making.

I use ‘currency’ because in its simplest sense, a currency is something of value agreed as a medium of exchange within a group. Differences in significance might be ascribed to it across groups; in early times, a cowrie shell might be an ornament for one tribe and a unit of currency for another. In scientific collaborations involving many disciplines and expertise communities, the ‘cowrie shell of data’ may not be the same thing for everyone; but it is often the object and initiator of exchanges from which meaning is sought and/or derived. Even if people disagree on the meaning of the data or the process of their collection, hammering out differences and co-designing plans to resolve differences begin the process of knowledge integration towards consilience. Data stimulate and ground our flights of imagination; and rigorous, sophisticated analysis of data can provide insights of pattern, order and prediction in the midst of seemingly overwhelming chaos. If properly generated and shared, data are the glue and currency of trust that bind collaboration.

So how we generate and make use of data is central to how we can successfully collaborate with each other to more effectively integrate knowledge from collective efforts. Experiments in science of learning across widely distributed locations have generated unprecedented volumes and types of data, including neuroimaging, electrophysiological recording, psychometric and behavioural, fine-structural and anatomic, genetic as well as learning data streams from computer tutoring systems. They bring to the fore the importance of capitalising on advances in technological tools to more effectively share, mine and analyse data; and to better understand the added value of data as potential catalysts and sources of new discovery and knowledge.

How do we add value to the data beyond their original purpose? A first step is to ensure their trustworthiness through quality and integrity of process by which they are generated. Foundational training in data literacy, data management, data sharing and ethical use of data is critical. Second, data become more valuable if they are understood and can be used by others. Again, training helps overcome ‘activation barriers’ and paves the way to cultivating practices that make fuller use of existing data. For example, preliminary analysis of sample datasets across disciplinary boundaries can identify key design principles and concepts to link studies and fill gaps of knowledge. Active re-use of data already collected refines practices within the community so that future producers of data are more likely to generate actionable data that are appropriately collected, annotated and securely stored. Third, there is a need to facilitate greater awareness of other data that could be harnessed for richer contextual framing of studies in learning (e.g., demographic data), with attendant emphasis on developing new analytical approaches to mine and interpret data; and fourth, we need to be creative about incentives and reward systems for data sharing, development of analytical tools and other resources of use to the community. Standards of practice for ensuring that the science is robust, reliable and transparent are critical for progress towards consilience.

Given the burgeoning interest in the science of learning among the researcher, practitioner and policy communities, it seems an opportune time to establish a ‘community of practice’. Such a community could jointly utilise existing and future data to create synthesis and solution of problems in scientific research, and to translate the research for educational use. ‘Communities of practice’ are defined as ‘groups of people who share a concern or a passion for something they do and learn how to do it better as they interact regularly’.^12^ Knowledge advancement through such ‘community’ participation is key for the goal of consilience and requires a change in the nature of work/discovery in ways that facilitate open access to knowledge, knowledge integration through collaboration and a commitment to the community.

The collaboration between Nature Publishing Group, the Queensland Brain Institute and Queensland University in launching the open access *Science of Learning* journal is an important step in this direction. With its emphasis on bringing together the findings of neuroscience, cognitive science and education research, the journal offers a useful forum for debate and discussion towards consilience in the science of learning. As Wilson^2^ stated, ‘the strongest appeal of consilience is in the prospect of intellectual adventure and, given even modest success, the value of understanding the human condition with a higher degree of certainty.’

*The views expressed in this article are those of the author. They do not necessarily represent the views of the National Science Foundation or the United States Government*.
